# Di-μ-bromido-bis­[(diethyl ether-κ*O*)(2,4,6-tri­methyl­phen­yl)magnesium]: the mesityl Grignard reagent

**DOI:** 10.1107/S1600536813017108

**Published:** 2013-06-29

**Authors:** Ömer Seven, Michael Bolte, Hans-Wolfram Lerner

**Affiliations:** a Institut für Anorganische und Analytische Chemie, Goethe-Universität Frankfurt, Max-von-Laue-Strasse 7, 60438 Frankfurt am Main, Germany

## Abstract

The crystal structure of the title compound, [Mg_2_Br_2_(C_9_H_11_)_2_(C_4_H_10_O)_2_], features a centrosymmetric two-centre magnesium complex with half a mol­ecule in the asymmetric unit. The Mg atom is in a considerably distorted Br_2_CO coordination. Bond lengths and angles are comparable with already published values. The crystal packing is stabilized by C—H⋯π inter­actions linking the complexes into sheets parallel to (0-11).

## Related literature

For literature on other Grignard reagents, see: Blasberg *et al.* (2012 ▶); Bock *et al.* (1996 ▶); Cole *et al.* (2003 ▶); Ellison & Power (1996 ▶); Hübner *et al.* (2010 ▶); Hayashi *et al.* (2011 ▶); Sakamoto *et al.* (2001 ▶); Waggoner & Power (1992 ▶).
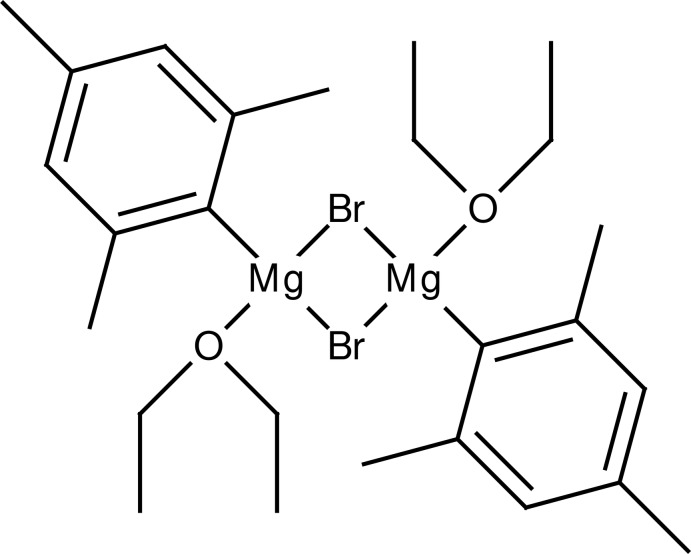


## Experimental

### 

#### Crystal data

[Mg_2_Br_2_(C_9_H_11_)_2_(C_4_H_10_O)_2_]
*M* *_r_* = 595.03
Triclinic, 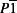

*a* = 7.8516 (6) Å
*b* = 8.8285 (6) Å
*c* = 12.1356 (8) Å
α = 87.285 (6)°
β = 82.516 (6)°
γ = 65.396 (5)°
*V* = 758.30 (10) Å^3^
*Z* = 1
Mo *K*α radiation
μ = 2.73 mm^−1^
*T* = 173 K
0.23 × 0.19 × 0.13 mm

#### Data collection

Stoe IPDS II two-circle diffractometer
Absorption correction: multi-scan (*X-AREA*; Stoe & Cie, 2001 ▶) *T*_min_ = 0.572, *T*_max_ = 0.718
18010 measured reflections
4227 independent reflections
3839 reflections with *I* > 2σ(*I*)
*R*_int_ = 0.048

#### Refinement

*R*[*F* ^2^ > 2σ(*F* ^2^)] = 0.036
*wR*(*F* ^2^) = 0.096
*S* = 1.06
4227 reflections
148 parameters
H-atom parameters constrained
Δρ_max_ = 0.70 e Å^−3^
Δρ_min_ = −0.55 e Å^−3^

### 

Data collection: *X-AREA* (Stoe & Cie, 2001 ▶); cell refinement: *X-AREA*; data reduction: *X-AREA*; program(s) used to solve structure: *SHELXS97* (Sheldrick, 2008 ▶); program(s) used to refine structure: *SHELXL2012* (Sheldrick, 2012 ▶); molecular graphics: *XP* (Sheldrick, 2008 ▶); software used to prepare material for publication: *PLATON* (Spek, 2009 ▶) and *publCIF* (Westrip, 2010 ▶).

## Supplementary Material

Crystal structure: contains datablock(s) I, global. DOI: 10.1107/S1600536813017108/ng5334sup1.cif

Structure factors: contains datablock(s) I. DOI: 10.1107/S1600536813017108/ng5334Isup2.hkl

Additional supplementary materials:  crystallographic information; 3D view; checkCIF report

## Figures and Tables

**Table:** 

Br1—Mg1	2.5503 (7)
Br1—Mg1^i^	2.5900 (7)
Mg1—O1	2.0243 (16)
Mg1—C11	2.123 (2)

**Table:** 

O1—Mg1—C11	108.18 (7)
O1—Mg1—Br1	105.96 (5)
C11—Mg1—Br1	121.22 (6)
O1—Mg1—Br1^i^	100.23 (5)
C11—Mg1—Br1^i^	126.30 (6)

Symmetry code: (i) 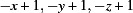.

**Table 2:** Hydrogen-bond geometry (Å, °) *Cg*1 is the centroid of the C1–C6 phenyl ring.

*D*—H⋯*A*	*D*—H	H⋯*A*	*D*—H⋯*A*
C3—H3*B*⋯*Cg*1^ii^	0.99	2.83	159
C18—H18*B*⋯*Cg*1^iii^	0.98	3.05	122

Symmetry codes: (ii) 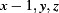; (iii) 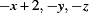.

**Fig. 1.**
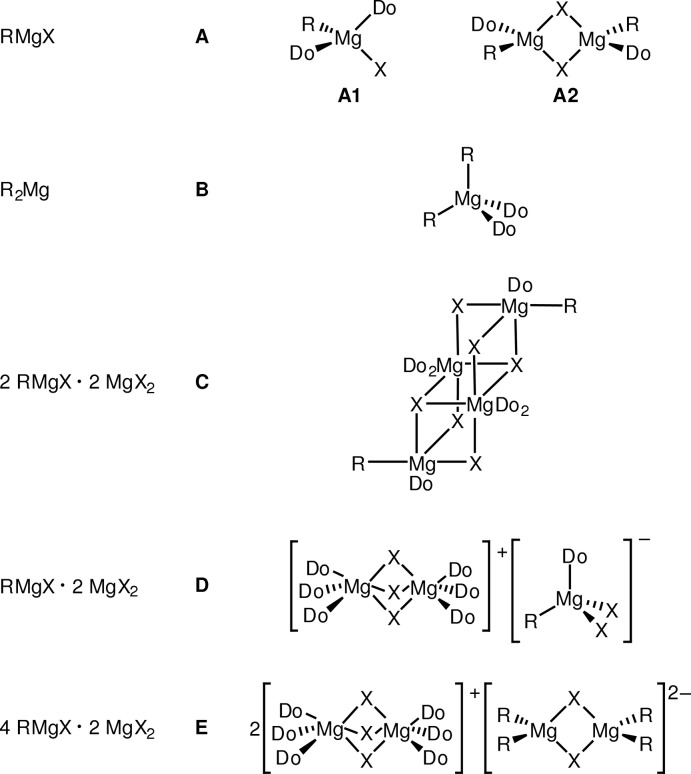
Structures of Magnesium Organyles in Donor Solvents (R = alkyl, aryl; X = halogen; Do = donor).

**Fig. 2.**
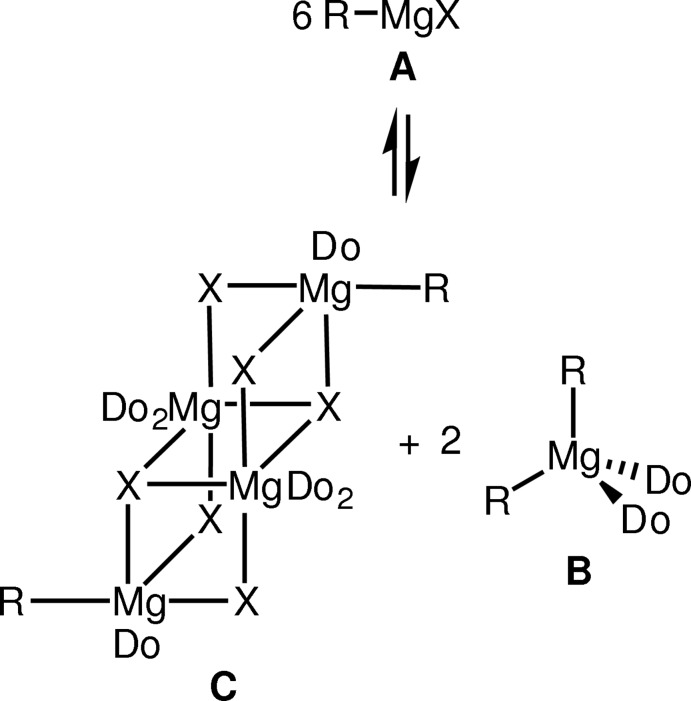
Grignard Compounds (RMgX) in Donor Solvents.

**Fig. 3.**
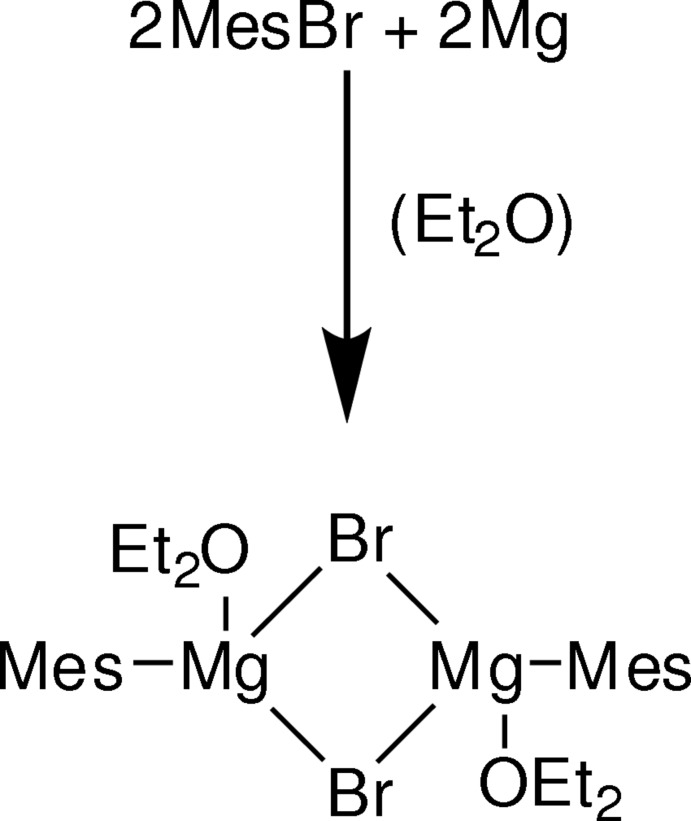
Synthesis of [MgBrMes(OEt2)]2.

**Fig. 4.**
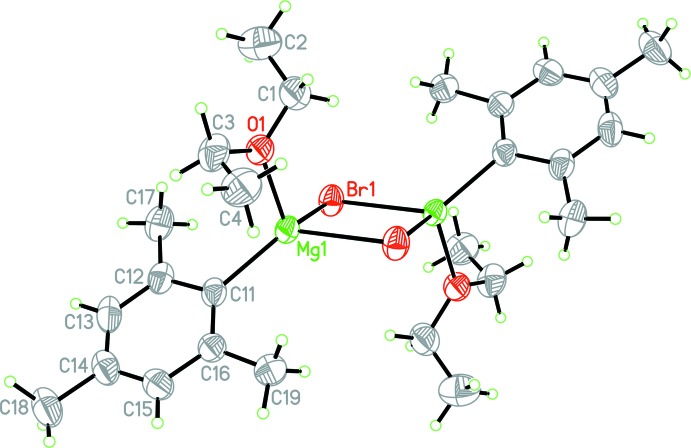
A perspective view of (I) showing the atom-numbering scheme. Displacement ellipsoids are drawn at the 50% probability level and H atoms are shown as small spheres of arbitrary radii. Only the symmetry independent atoms have been labelled. Symmetry equivalent atoms were generated applying the symmetry operator -x + 1, -y + 1, -z + 1.

**Fig. 5.**
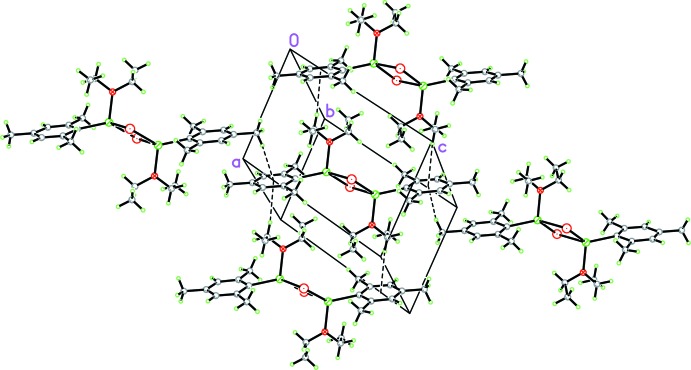
Crystal packing of (I) showing the C—H···cog interactions as dashed lines.

**Fig. 6.**
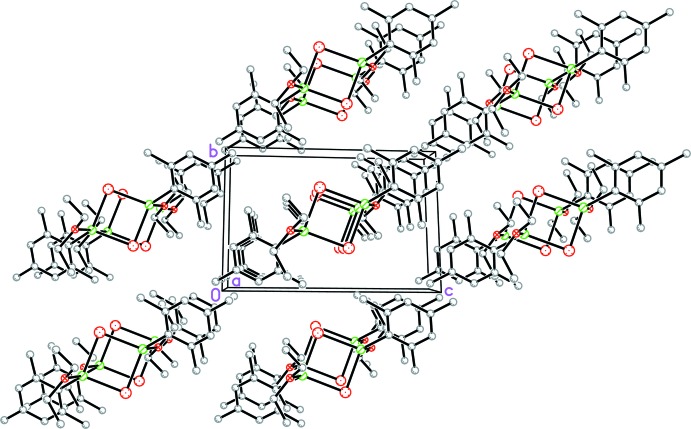
Crystal packing of (I) with view along the a axis showing the sheets of molecules.

## supplementary crystallographic information

### Comment

Besides organo lithium compounds Grignard reagents *R*Mg*X* are among the most widely used metal organic reagents in synthesis (Figure 1). It is therefore not surprising that much effort has been devoted to structural elucidation both in the solid state and in solution, since knowing the structure of highly reactive reagents is of essential importance, when it comes to understanding the principles governing the high reactivity. When the structural characteristics of Grignard reagents are understood, tuning of their reactivity becomes possible. It was reported (Sakamoto *et al.*, 2001; Blasberg *et al.*, 2012) that there is an equilibrium between the Grignard compounds *R*Mg*X* on the one hand and diorganylmagnesium B and the Mg*X*_2_ adducts C on the other hand (Figure 2). This finding can be regarded as an extension of the Schlenk equilibrium. However, in same cases magnesate complexes of type D and E were isolated from Grignard solution.

The mesityl Grignard reagent MgBrMes established itself as a practical and easily accessible nucleophile. As a unique building block, MgBrMes has been used in various reactions in the last a few decades. The commercial use is justified on the one hand by the *C*_2_ symmetry and on the other hand by the increased steric bulk at the 2,6-positions of the mesityl unit. Compared to phenyl Grignard reagent, the three methyl groups of MgBrMes increase the solubility of the product yielding from a reaction with an electrophile. Furthermore, the three methyl groups of the mesityl ring facilitate analytical investigations of obtained products: for instance, in a ^1^H NMR spectrum, the integral ratio between the methyl groups of the mesityl unit and the proton of the electrophile gives a well defined product.

Therefore the structural elucidation of MgBrMes is of great interest. We obtained X-ray quality crystals of [MgBrMes(OEt_2_)]_2_ by gas diffusion of toluene in the filtered diethyl ether reaction solution of MgBrMes and *ortho*-C_6_H_4_(BH_2_(pyridine))_2_. As shown in Figure 3, the Grignard compound MgBrMes was thereby synthesized by a textbook procedure.

In contrast to the structure of mesityl lithium (LiMes) which crystallizes as an infinite zigzag-chain of [LiMes]_2_ dimers along the crystallographic *c* axis (Hübner *et al.*, 2010) the crystal structure of [MgBrMes(OEt_2_)]_2_ reveals discrete dimers of the type A2 in the solid state and no magnesates of type C, D, and E were crystallized.

The structural parameters of the mesityl anion in [MgBrMes(OEt_2_)]_2_ are comparable with those found in the corresponding homoleptic compounds [LiMes]_2_ (Hübner *et al.*, 2010) and [MMes_2_] (*M* = Mg, Zn, Cd, Hg; Waggoner & Power, 1992; Cole *et al.*, 2003; Hayashi *et al.*, 2011).

The title compound features a centrosymmetric two-centre magnesium complex (Figure 4). There is just half a molecule in the asymmetric unit. Each Mg centre is four-coordinated by two bridging bromo atoms, one oxygen atom of a diethyl ether molecule and a carbon atom of a mesityl ligand. The coordination sphere of the Mg centres is a distorted tetrahedron (Table 1). The two Mg—Br bonds show essentially the same length. The bond angles at Mg range from 91.72 (2)° for Br—Mg—Br to 126.30 (6)° for C—Mg—Br. Whereas the innercyclic Br—Mg—Br angle is significantly smaller than the ideal tetrahedral value, the two C—Mg—Br angles are substantially widened. The bond angle at Br is 88.28 (2)°. The dihedral angle between the four-membered Mg_2_Br_2_ ring and the aromatic ring is 40.39 (4)°. It is remarkable to note that none of the ether methyl groups adopts an antiperiplanar conformation with respect to the Mg centre (Table 1). The torsion angle C4—C3—O1—Mg1 adopts a *gauche* conformation and the torsion angle C2—C3—O1—Mg1 adopts a partially eclipsed conformation.

A search in the Cambridge Crystallographic Database yielded only two structures with a four-coordinated Mg centre bonded to two bromo atoms, an oxygen atom and an aromatic carbon atom, which are bis((µ_2_-bromo)-(dibutyl ether)-anthracen-9-yl-magnesium) (CSD refcode TATNAD; Bock *et al.*, 1996) and bis((µ_2_-bromo)-(2,6-dimesitylphenyl)-(tetrahydrofuran)-magnesium) (CSD refcode RUGNEM; Ellison & Power, 1996). Thus, average bond distances for a four-coordinated Mg atom to a three-coordinated O atom, a four-coordinated Mg atom to an aromatic C atom, and a four-coordinated Mg atom to a two-coordinated Br atom were retrieved from three different searches in the CSD (Table 2). These values agree well with those of the title compound and the two already retrieved structures having a four-coordinated Mg centre bonded to two bromo atoms, an oxygen atom and an aromatic carbon atom.

The crystal packing of the title compound (Figure 5) shows that the molecules are connected by C—H···π interactions. One is between a methylene group at C3 and the centroid (cog) of a mesityl ring of a neighbouring molecule (C3—H3B 0.990 Å, H3B···cog^i^ 2.833 Å, C3—H3B···cog^i^ 158.9°; symmetry operator (i) *x* - 1, *y*, *z*). The other one connects a methyl group at C18 and the centroid of a mesityl ring of a another molecule (C18—H18B 0.980 Å, H18B···cog^ii^ 3.047 Å, C3—H3B···cog^ii^ 122.1°; symmetry operator (ii) -*x* + 2, -*y*, -*z*). These interactions connect the complexes to sheets parallel to the (0 1 1) plane (Figure 6).

### Experimental

MgBrMes: In a two-necked flask equipped with a reflux condenser and a dropping funnel, Mg turnings (1.0 g, 41.1 mmol) were heated with a heat gun under vacuum for 15 min. After the flask had cooled to room temperature again, diethyl ether (50 ml) was added. A solution of 2-bromomesitylene (5.0 ml, 6.5 g, 32.7 mmol) in diethyl ether (10 ml) was added dropwise over 1 h to the vigorously stirred reaction mixture. After the addition was complete, the yellowish brown reaction mixture was heated at reflux temperature for 8 h and allowed to cool to room temperature again. The mixture was filtered, and the concentration of the filtrate was determined by titration (0.38 *M*). Single crystals of [MgBrMes(OEt_2_)]_2_ were obtained by the following procedure: In a two-necked flask equipped with a reflux condenser and a dropping funnel, the pyridine adduct with ditopic borane *ortho*-C_6_H_4_(BH_2_(pyridine))_2_ (1.1 g, 4.2 mmol) was suspended in 20 ml diethyl ether. The mesityl Grignard reagent (0.38 *M* in diethyl ether, 22 ml, 8.4 mmol) was added dropwise over 3 h to the yellowish suspension. After the addition was complete, the reddish suspension was heated 1 h at reflux temperature and finally the formed precipitate was removed by filtration. Gas diffusion of toluene into the filtrate gave colorless crystals of [MgBrMes(OEt_2_)]_2_.

### Refinement

All H atoms were located in difference Fourier maps. Nevertheless, they were geometrically positioned and refined using a riding model with aromatic C—H = 0.95 Å, methyl C—H = 0.98 Å, secondary C—H = 0.99 Å, and with *U*_iso_(H) = 1.5*U*_eq_(C) for methyl H or 1.2*U*_eq_(C) for secondary and aromatic H. The methyl groups of the mesityl rings were allowed to rotate but not to tip.

### Crystal data

**Table:**

[Mg_2_Br_2_(C_9_H_11_)_2_(C_4_H_10_O)_2_]	*Z* = 1
*M**_r_* = 595.03	*F*(000) = 308
Triclinic, *P*1	*D*_x_ = 1.303 Mg m^−^^3^
*a* = 7.8516 (6) Å	Mo *K*α radiation, λ = 0.71073 Å
*b* = 8.8285 (6) Å	Cell parameters from 29361 reflections
*c* = 12.1356 (8) Å	θ = 3.4–30.1°
α = 87.285 (6)°	µ = 2.73 mm^−^^1^
β = 82.516 (6)°	*T* = 173 K
γ = 65.396 (5)°	Plate, colourless
*V* = 758.30 (10) Å^3^	0.23 × 0.19 × 0.13 mm

### Data collection

**Table:**

Stoe IPDS II two-circle diffractometer	3839 reflections with *I* > 2σ(*I*)
Radiation source: Genix 3D IµS microfocus X-ray source	*R*_int_ = 0.048
ω scans	θ_max_ = 29.7°, θ_min_ = 3.2°
Absorption correction: multi-scan (*X-AREA*; Stoe & Cie, 2001)	*h* = −10→10
*T*_min_ = 0.572, *T*_max_ = 0.718	*k* = −12→12
18010 measured reflections	*l* = −16→14
4227 independent reflections	

### Refinement

**Table:**

Refinement on *F*^2^	0 restraints
Least-squares matrix: full	Hydrogen site location: inferred from neighbouring sites
*R*[*F*^2^ > 2σ(*F*^2^)] = 0.036	H-atom parameters constrained
*wR*(*F*^2^) = 0.096	*w* = 1/[σ^2^(*F*_o_^2^) + (0.0555*P*)^2^ + 0.1885*P*] where *P* = (*F*_o_^2^ + 2*F*_c_^2^)/3
*S* = 1.06	(Δ/σ)_max_ = 0.001
4227 reflections	Δρ_max_ = 0.70 e Å^−^^3^
148 parameters	Δρ_min_ = −0.55 e Å^−^^3^

### Special details

**Table:**

Geometry. All e.s.d.'s (except the e.s.d. in the dihedral angle between two l.s. planes) are estimated using the full covariance matrix. The cell e.s.d.'s are taken into account individually in the estimation of e.s.d.'s in distances, angles and torsion angles; correlations between e.s.d.'s in cell parameters are only used when they are defined by crystal symmetry. An approximate (isotropic) treatment of cell e.s.d.'s is used for estimating e.s.d.'s involving l.s. planes.

### Fractional atomic coordinates and isotropic or equivalent isotropic displacement parameters (Å^2^)

**Table:**

	*x*	*y*	*z*	*U*_iso_*/*U*_eq_	
Br1	0.47162 (3)	0.70177 (2)	0.43643 (2)	0.04331 (8)	
Mg1	0.56669 (9)	0.40755 (8)	0.36418 (5)	0.03510 (14)	
O1	0.3353 (2)	0.4088 (2)	0.30685 (13)	0.0454 (3)	
C1	0.1452 (4)	0.4898 (4)	0.3613 (2)	0.0646 (7)	
H1A	0.1459	0.5477	0.4290	0.077*	
H1B	0.0966	0.4047	0.3844	0.077*	
C2	0.0172 (5)	0.6124 (4)	0.2880 (3)	0.0745 (8)	
H2A	−0.1102	0.6646	0.3281	0.112*	
H2B	0.0139	0.5553	0.2215	0.112*	
H2C	0.0634	0.6981	0.2660	0.112*	
C3	0.3630 (4)	0.2712 (3)	0.2340 (2)	0.0549 (6)	
H3A	0.4537	0.2676	0.1682	0.066*	
H3B	0.2416	0.2902	0.2077	0.066*	
C4	0.4358 (4)	0.1090 (4)	0.2927 (3)	0.0650 (7)	
H4A	0.4529	0.0192	0.2420	0.097*	
H4B	0.3452	0.1118	0.3572	0.097*	
H4C	0.5571	0.0891	0.3177	0.097*	
C11	0.8009 (3)	0.2966 (3)	0.24069 (16)	0.0376 (4)	
C12	0.8019 (3)	0.3733 (3)	0.13610 (18)	0.0435 (4)	
C13	0.9428 (4)	0.2965 (4)	0.04831 (19)	0.0517 (5)	
H13	0.9401	0.3527	−0.0206	0.062*	
C14	1.0863 (3)	0.1401 (3)	0.0595 (2)	0.0509 (5)	
C15	1.0878 (3)	0.0633 (3)	0.1619 (2)	0.0479 (5)	
H15	1.1845	−0.0441	0.1716	0.057*	
C16	0.9509 (3)	0.1396 (3)	0.25080 (18)	0.0404 (4)	
C17	0.6470 (4)	0.5430 (3)	0.1164 (2)	0.0579 (6)	
H17A	0.6757	0.5828	0.0425	0.087*	
H17B	0.6391	0.6219	0.1731	0.087*	
H17C	0.5260	0.5339	0.1207	0.087*	
C18	1.2346 (4)	0.0568 (5)	−0.0371 (3)	0.0665 (8)	
H18A	1.3152	0.1171	−0.0534	0.100*	
H18B	1.1729	0.0580	−0.1025	0.100*	
H18C	1.3118	−0.0586	−0.0178	0.100*	
C19	0.9662 (4)	0.0501 (3)	0.3609 (2)	0.0531 (5)	
H19A	0.8588	0.0200	0.3788	0.080*	
H19B	0.9656	0.1233	0.4193	0.080*	
H19C	1.0840	−0.0512	0.3560	0.080*	

### Atomic displacement parameters (Å^2^)

**Table:**

	*U*^11^	*U*^22^	*U*^33^	*U*^12^	*U*^13^	*U*^23^
Br1	0.05753 (14)	0.03546 (11)	0.03474 (11)	−0.01729 (8)	−0.00527 (8)	0.00114 (7)
Mg1	0.0392 (3)	0.0381 (3)	0.0299 (3)	−0.0168 (3)	−0.0070 (2)	−0.0023 (2)
O1	0.0436 (7)	0.0608 (9)	0.0388 (7)	−0.0271 (7)	−0.0068 (6)	−0.0099 (6)
C1	0.0522 (14)	0.096 (2)	0.0501 (14)	−0.0355 (14)	−0.0017 (11)	−0.0116 (14)
C2	0.0584 (16)	0.0700 (18)	0.096 (3)	−0.0244 (14)	−0.0198 (16)	−0.0042 (17)
C3	0.0679 (15)	0.0638 (14)	0.0474 (12)	−0.0386 (12)	−0.0135 (11)	−0.0086 (10)
C4	0.0670 (16)	0.0671 (16)	0.0712 (18)	−0.0358 (14)	−0.0158 (14)	0.0015 (14)
C11	0.0418 (9)	0.0448 (9)	0.0318 (9)	−0.0230 (8)	−0.0050 (7)	−0.0035 (7)
C12	0.0525 (11)	0.0531 (11)	0.0341 (9)	−0.0302 (9)	−0.0079 (8)	0.0011 (8)
C13	0.0634 (14)	0.0732 (15)	0.0318 (9)	−0.0425 (12)	−0.0006 (9)	−0.0049 (9)
C14	0.0490 (12)	0.0728 (15)	0.0440 (12)	−0.0389 (11)	0.0046 (9)	−0.0200 (10)
C15	0.0406 (10)	0.0548 (12)	0.0515 (12)	−0.0225 (9)	−0.0015 (9)	−0.0166 (10)
C16	0.0396 (9)	0.0466 (10)	0.0389 (10)	−0.0208 (8)	−0.0056 (8)	−0.0051 (8)
C17	0.0731 (16)	0.0603 (14)	0.0420 (12)	−0.0291 (12)	−0.0121 (11)	0.0120 (10)
C18	0.0574 (14)	0.098 (2)	0.0557 (15)	−0.0463 (15)	0.0119 (12)	−0.0291 (14)
C19	0.0505 (12)	0.0512 (12)	0.0490 (13)	−0.0127 (10)	−0.0075 (10)	0.0047 (10)

### Geometric parameters (Å, º)

**Table:**

Br1—Mg1	2.5503 (7)	C11—C12	1.411 (3)
Br1—Mg1^i^	2.5900 (7)	C12—C13	1.399 (3)
Mg1—O1	2.0243 (16)	C12—C17	1.518 (4)
Mg1—C11	2.123 (2)	C13—C14	1.386 (4)
Mg1—Br1^i^	2.5900 (7)	C13—H13	0.9500
O1—C1	1.440 (3)	C14—C15	1.385 (4)
O1—C3	1.462 (3)	C14—C18	1.511 (3)
C1—C2	1.486 (5)	C15—C16	1.392 (3)
C1—H1A	0.9900	C15—H15	0.9500
C1—H1B	0.9900	C16—C19	1.513 (3)
C2—H2A	0.9800	C17—H17A	0.9800
C2—H2B	0.9800	C17—H17B	0.9800
C2—H2C	0.9800	C17—H17C	0.9800
C3—C4	1.488 (4)	C18—H18A	0.9800
C3—H3A	0.9900	C18—H18B	0.9800
C3—H3B	0.9900	C18—H18C	0.9800
C4—H4A	0.9800	C19—H19A	0.9800
C4—H4B	0.9800	C19—H19B	0.9800
C4—H4C	0.9800	C19—H19C	0.9800
C11—C16	1.409 (3)		
			
Mg1—Br1—Mg1^i^	88.28 (2)	H4B—C4—H4C	109.5
O1—Mg1—C11	108.18 (7)	C16—C11—C12	115.81 (19)
O1—Mg1—Br1	105.96 (5)	C16—C11—Mg1	124.39 (15)
C11—Mg1—Br1	121.22 (6)	C12—C11—Mg1	119.46 (16)
O1—Mg1—Br1^i^	100.23 (5)	C13—C12—C11	121.6 (2)
C11—Mg1—Br1^i^	126.30 (6)	C13—C12—C17	118.3 (2)
Br1—Mg1—Br1^i^	91.72 (2)	C11—C12—C17	120.1 (2)
O1—Mg1—Mg1^i^	108.92 (5)	C14—C13—C12	121.5 (2)
C11—Mg1—Mg1^i^	142.89 (6)	C14—C13—H13	119.3
Br1—Mg1—Mg1^i^	46.315 (16)	C12—C13—H13	119.3
Br1^i^—Mg1—Mg1^i^	45.403 (16)	C15—C14—C13	117.6 (2)
C1—O1—C3	113.41 (19)	C15—C14—C18	121.5 (3)
C1—O1—Mg1	124.89 (15)	C13—C14—C18	120.8 (3)
C3—O1—Mg1	117.26 (15)	C14—C15—C16	121.6 (2)
O1—C1—C2	112.1 (3)	C14—C15—H15	119.2
O1—C1—H1A	109.2	C16—C15—H15	119.2
C2—C1—H1A	109.2	C15—C16—C11	121.9 (2)
O1—C1—H1B	109.2	C15—C16—C19	118.4 (2)
C2—C1—H1B	109.2	C11—C16—C19	119.79 (19)
H1A—C1—H1B	107.9	C12—C17—H17A	109.5
C1—C2—H2A	109.5	C12—C17—H17B	109.5
C1—C2—H2B	109.5	H17A—C17—H17B	109.5
H2A—C2—H2B	109.5	C12—C17—H17C	109.5
C1—C2—H2C	109.5	H17A—C17—H17C	109.5
H2A—C2—H2C	109.5	H17B—C17—H17C	109.5
H2B—C2—H2C	109.5	C14—C18—H18A	109.5
O1—C3—C4	111.2 (2)	C14—C18—H18B	109.5
O1—C3—H3A	109.4	H18A—C18—H18B	109.5
C4—C3—H3A	109.4	C14—C18—H18C	109.5
O1—C3—H3B	109.4	H18A—C18—H18C	109.5
C4—C3—H3B	109.4	H18B—C18—H18C	109.5
H3A—C3—H3B	108.0	C16—C19—H19A	109.5
C3—C4—H4A	109.5	C16—C19—H19B	109.5
C3—C4—H4B	109.5	H19A—C19—H19B	109.5
H4A—C4—H4B	109.5	C16—C19—H19C	109.5
C3—C4—H4C	109.5	H19A—C19—H19C	109.5
H4A—C4—H4C	109.5	H19B—C19—H19C	109.5
			
C3—O1—C1—C2	−81.3 (3)	C12—C13—C14—C15	−1.3 (3)
Mg1—O1—C1—C2	123.2 (2)	C12—C13—C14—C18	178.4 (2)
C1—O1—C3—C4	−94.6 (3)	C13—C14—C15—C16	−0.2 (3)
Mg1—O1—C3—C4	62.9 (3)	C18—C14—C15—C16	−179.8 (2)
C16—C11—C12—C13	0.5 (3)	C14—C15—C16—C11	1.8 (3)
Mg1—C11—C12—C13	−173.11 (16)	C14—C15—C16—C19	−178.0 (2)
C16—C11—C12—C17	−179.8 (2)	C12—C11—C16—C15	−1.9 (3)
Mg1—C11—C12—C17	6.6 (3)	Mg1—C11—C16—C15	171.33 (15)
C11—C12—C13—C14	1.1 (3)	C12—C11—C16—C19	177.86 (19)
C17—C12—C13—C14	−178.6 (2)	Mg1—C11—C16—C19	−8.9 (3)

Symmetry code: (i) −*x*+1, −*y*+1, −*z*+1.

### Hydrogen-bond geometry (Å, º)

Cg1 is the centroid of the C1–C6 phenyl ring.

**Table:**

*D*—H···*A*	*D*—H	H···*A*	*D*—H···*A*
C3—H3*B*···*Cg*1^ii^	0.99	2.83	159
C18—H18*B*···*Cg*1^iii^	0.98	3.05	122

Symmetry codes: (ii) *x*−1, *y*, *z*; (iii) −*x*+2, −*y*, −*z*.

### Bond distances (Å) involving the Mg centre

**Table:**

	Mg—O	Mg—C	Mg—Br	Mg—Br
Title compound	2.0243 (16)	2.123 (2)	2.5503 (7)	2.5900 (7)
RUGNEM	2.011	2.131	2.558	2.580
TATNAD	2.024	2.130	2.572	2.582
CSD	2.02 (6)	2.17 (5)	2.57 (8)	

## References

[bb1] Blasberg, F., Bolte, M., Wagner, M. & Lerner, H.-W. (2012). *Organometallics*, **31**, 1001–1005.

[bb2] Bock, H., Ziemer, K. & Näther, C. (1996). *J. Organomet. Chem.* **511**, 29–35.

[bb3] Cole, S. C., Coles, M. P. & Hitchcock, P. B. (2003). *Dalton Trans.* pp. 3663–3664.

[bb4] Ellison, J. J. & Power, P. P. (1996). *J. Organomet. Chem.* **526**, 263–267.

[bb5] Hayashi, M., Bolte, M., Wagner, M. & Lerner, H.-W. (2011). *Z. Anorg. Allg. Chem.* **637**, 646–649.

[bb6] Hübner, A., Bernert, T., Sänger, I., Alig, E., Bolte, M., Fink, L., Wagner, M. & Lerner, H.-W. (2010). *Dalton Trans.* **39**, 7528–7533. 10.1039/c0dt00161a 20614079

[bb7] Sakamoto, S., Imamoto, T. & Yamaguchi, K. (2001). *Org. Lett.* **3**, 1793–1795. 10.1021/ol010048x 11405713

[bb8] Sheldrick, G. M. (2008). *Acta Cryst.* A**64**, 112–122. 10.1107/S0108767307043930 18156677

[bb9] Sheldrick, G. M. (2012). *SHELXL2012* University of Göttingen, Germany.

[bb10] Spek, A. L. (2009). *Acta Cryst.* D**65**, 148–155. 10.1107/S090744490804362X PMC2631630 19171970

[bb11] Stoe & Cie (2001). *X-AREA* Stoe & Cie, Darmstadt, Germany.

[bb12] Waggoner, K. M. & Power, P. P. (1992). *Organometallics*, **11**, 3209–3214.

[bb13] Westrip, S. P. (2010). *J. Appl. Cryst.* **43**, 920–925.

